# Assessing the Aging Effect on Ti/Au Bilayers for Transition-Edge Sensor (TES) Detectors

**DOI:** 10.3390/s24123995

**Published:** 2024-06-20

**Authors:** Maria Gambelli, Matteo D’Andrea, Rita Asquini, Alessio Buzzin, Claudio Macculi, Guido Torrioli, Sara Cibella

**Affiliations:** 1 Institute for Photonics and Nanotechnologies, National Research Council of Italy (CNR), Via del Fosso del Cavaliere 100, 00133 Rome, Italy; guido.torrioli@ifn.cnr.it (G.T.); sara.cibella@ifn.cnr.it (S.C.); 2 Institute of Space Astrophysics and Planetology, Italian National Institute for Astrophysics (INAF), Via del Fosso del Cavaliere 100, 00133 Rome, Italy; matteo.dandrea@inaf.it (M.D.); claudio.macculi@inaf.it (C.M.); 3 Department of Information Engineering, Electronics and Telecommunications, Via Eudossiana 18, Sapienza University of Rome, 00184 Rome, Italy; rita.asquini@uniroma1.it (R.A.); alessio.buzzin@uniroma1.it (A.B.)

**Keywords:** TES, Ti/Au, aging effect, proximity effect, annealing, superconductivity

## Abstract

Transition-edge sensor (TES) microcalorimeters are advanced cryogenic detectors that use a superconducting film for particle or photon detection. We are establishing a new production line for TES detectors to serve as cryogenic anticoincidence (i.e., veto) devices. These detectors are made with a superconducting bilayer of titanium (Ti) and gold (Au) thin films deposited via electron beam evaporation in a high vacuum condition on a monocrystalline silicon substrate. In this work, we report on the development of such sensors, aiming to achieve stable sensing performance despite the effects of aging. For this purpose, patterned and non-patterned Ti/Au bilayer samples with varying geometries and thicknesses were fabricated using microfabrication technology. To characterize the detectors, we present and discuss initial results from repeated resistance–temperature (R–T) measurements over time, conducted on different samples, thereby augmenting existing literature data. Additionally, we present a discussion of the sensor’s degradation over time due to aging effects and test a potential remedy based on an easy annealing procedure. In our opinion, this work establishes the groundwork for our new TES detector production line.

## 1. Introduction

Transition-edge sensor (TES) microcalorimeters are ultra-sensitive cryogenic detectors for photon and particle detection. They operate based on a superconducting film that is biased within its superconducting-to-normal transition region. TESs are well-known due to their high quantum efficiency and remarkable energy resolution (∼2 eV at 6 keV), representing a cutting-edge technology suitable for many fields, from X-ray detection to fundamental physics (see [1] and references therein). In most of these applications, the particle background, i.e., the noise with respect to the target particle, is typically regarded as a major critical issue affecting the device’s sensitivity. Specific techniques have been applied to reduce the particle background, such as the association of active anticoincidence (i.e., veto) devices with TES-based main detectors [2]. As devices close (∼1 mm) to the main detector, technologically compatible anticoincidence devices offer interesting features, enabling a higher degree of integration thanks to better thermal and mechanical interfaces and allowing for simplification of the readout electronics [2,3].

TES detectors are made up of three main elements: an absorber layer, where particles or photons release their energy, a sensitive element acting as a thermometer and a thermal link connecting the absorber layer to a thermal bath. Our work is focused on a TES detector whose absorber layer is a monocrystalline silicon substrate, and the sensor element is a thin-film bilayer built directly over the substrate through microfabrication processes. In the bilayer, the normal-to-superconducting transition occurs at the critical temperature $T_{C}$, which depends on the superconducting material. The critical temperature is one of the most relevant TES features, since it strongly influences the detector properties, such as heat capacity *C* or thermal conductance *G*, and governs the most relevant detector performance parameters (energy bandwidth, characteristic response time, and energy resolution) [4]. The bilayer is usually made of a superconducting and a normal metal film, in order to fine-tune the $T_{c}$ by exploiting the proximity effect [4]. In this case, the critical temperature can be expressed as a function of normal metal ($d_{n}$) and superconductor ($d_{s}$) thicknesses through the model based on Usadel equations and developed by Martinis et al. [5]. Among all the possible choices as superconducting/normal bilayers, the use of molybdenum/gold (Mo/Au) [6,7,8,9], titanium/gold (Ti/Au) [10,11], and iridium/gold (Ir/Au) [12] are often reported. Moreover, tungsten monolayers (W TES) have been also used for this purpose [13].

In making our particle sensor, we used a thin-film Ti/Au bilayer, since it has the advantage of being compatible with the conventional technique of micro- and nanofabrication, allowing for a simple manufacturing process [14]. Nonetheless, as it is widely reported in the literature [14,15,16,17,18,19,20], a common issue with such Ti/Au film bilayers and Ti-only films is the critical temperature instability over time as a result of aging phenomena, which strongly affect the overall detector performances. In this work, we report the development of Ti/Au bilayers for TES detectors to be used as a cryogenic anticoincidence device. Ti/Au bilayers have been fabricated, together with Ti monolayers, and their resistance–temperature curves have been characterized in a cryogenic environment. The aging effect has been assessed through a systematic study on samples with different thicknesses and in different storage scenarios, aiming to extend the data already reported in the literature. The sample’s performance decrease has been studied and fitted with a degradation model in order to predict the aging effect. Finally, an annealing procedure has been introduced based on the literature and effectively enforced to fix the bilayers’ performances over time.

## 2. Materials and Methods

Ti/Au samples were fabricated with two purposes: non-patterned Ti/Au bilayer samples were characterized to assess and optimize the bilayer properties, while patterned Ti/Au bilayer samples were evaluated as the actual sensitive element of the TES detectors.

### 2.1. Patterned Bilayer Samples

**Figure 1:**
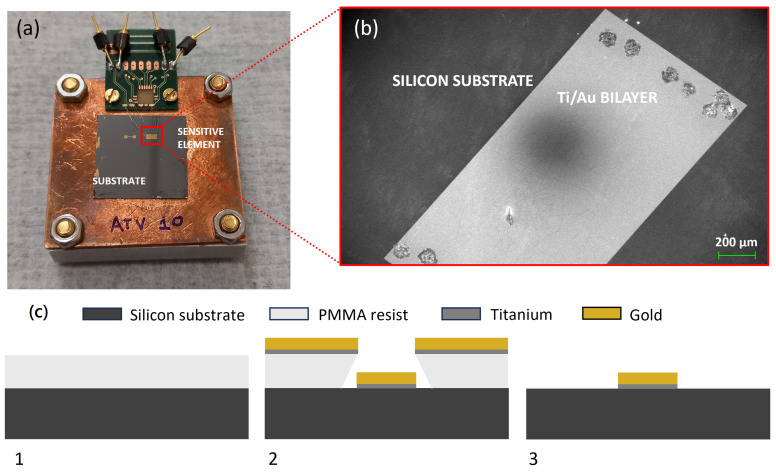
(**a**) Picture of patterned Ti/Au bilayer samples. (**b**) SEM enlargement of a Ti/Au bilayer two-square geometry. (**c**) 2D cross-section schematic sketch of fabrication steps for a patterned Ti/Au sample: spin coating of PMMA resist on a single crystal silicon substrate ready for EBL exposure (**1**), PMMA development after EBL exposure followed by Ti/Au e-beam evaporation (**2**), and lift-off with acetone (**3**).

Two different geometries achieved through microfabrication technologies are reported here. The fabrication steps are schematically depicted in Figure 1c.

Monocrystalline silicon substrates were cleaned with acetone and isopropanol. Electron beam lithography (Raith VOYAGER lithography system) on PMMA e-resist was used to define the two square geometries: the (1 × 2) mm^2^ rectangular structure (Figure 1a, right pattern) and the “strip” geometry, consisting of a (1 × 0.1) mm^2^ strip with two (0.6 × 0.6) mm^2^ lateral pads for wired connections, with a total footprint equal to (0.6 × 2.2) mm^2^ (Figure 1a, left pattern). The Ti/Au bilayer is then deposited by electron beam evaporation in high vacuum conditions without substrate heating. A Balzers e-beam evaporation system is used, with a base pressure under $3\times 10^{-7}$ mbar, ensuring the deposition of both Ti and Au films without exposing the Ti film to air. The two films are deposited in rapid succession, taking a time interval of 2 min to switch the evaporation procedure for the two samples. To increase the protection of the samples’ purity from gaseous contaminants (such as oxygen molecules) during the deposition, a 70 nm thick titanium layer is evaporated before the actual Ti/Au deposition on the substrates [21]. The Ti and Au films were deposited at a rate of 1.5 Å/s and 0.5 Å/s, respectively. The starting pressure for Ti deposition was $2.2\times 10^{-7}$ mbar. The thickness of the Ti/Au layers was controlled through a high-precision quartz micro-balance (QMC). As the last step, a lift-off procedure was performed using acetone in an ultrasound bath to achieve the desired patterns. Both patterns come from the same deposited Ti/Au stack, with thicknesses equal to 90 nm and 60 nm, respectively.

Two patterned samples, named ATV10 and ATV11, were fabricated with the same Ti/Au 90/60 nm thick bilayer but in two different fabrication runs. In particular, for the ATV10 sample, a preliminary cleaning procedure using the Reactive Ion Etching (RIE) technique based on oxygen plasma was performed on the bare silicon substrate.

Figure 1a shows the two patterned geometries at the end of the fabrication procedure on the same silicon substrate, and Figure 1b shows a SEM picture of the two-square pattern (see Section 3.1).

### 2.2. Non-Patterned Samples

For the non-patterned samples, the lithographic step was skipped, and the Ti/Au thin film bilayer was deposited directly on the monocrystalline silicon substrate following the same procedure as described in Section 2.1 for patterned samples.

The non-patterned samples were fabricated at different thicknesses: 90 nm/60 nm thick (Au/Ti ratio of 0.67), 90 nm/120 nm thick (Au/Ti ratio of 1.33), 60 nm/90 nm thick (Au/Ti ratio of 1.5). Lastly, a 90 nm thick Ti film was deposited to act as a reference.

## 3. Characterization

**Figure 2:**
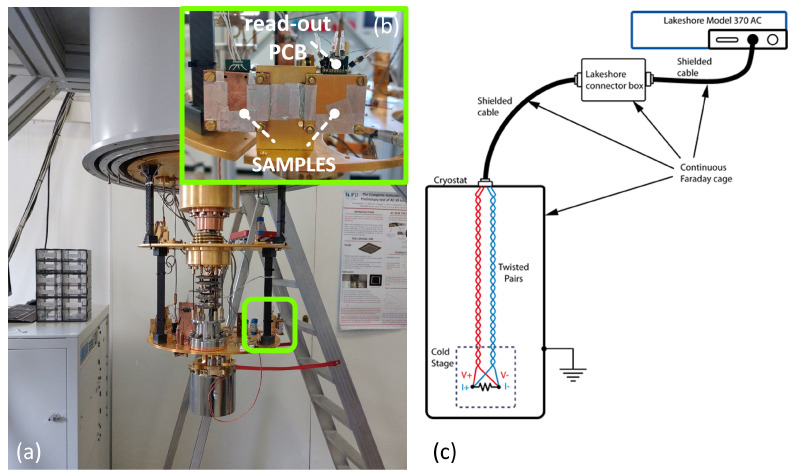
Measurement setup: (**a**) ^3^He/^4^He dilution cryostat by Oxford Instruments, circled in green, and sample holder anchored on the lowest temperature plate of the cryostat (see (**b**)). (**b**) Sample holder detail: PCB for 4-wire read out, and two samples enclosed between copper sheets. (**c**) Complete measurement setup: the LakeShore 370 AC Resistance Bridge connected to the cryostat through shielded cables. All the setup components form a continuous Faraday cage.

The samples were electrically characterized by measuring the resistance–temperature trend. First, a cryostat (^3^He/^4^He dilution cryostat by Oxford Instruments, see Figure 2a) was used to cool down the samples, reaching a base temperature of 10 mK. The entire cooling process took about 24 h. Inside the cryostat, the samples were enclosed between two copper sheets to avoid radioactive loads and fixed with rubber cement glue to allow for good thermalization (see Figure 2b) [22]. A dedicated printed circuit board (PCB) was fixed between the two sheets, allowing read out of the resistance using the 4-wire method, with the help of a resistance bridge (LakeShore, Model 370 AC) in current excitation mode. The excitation current has been kept low ($I_{exc}$ = 1 μA) to ensure that the dissipated power is in the range of a few pW, thus preventing the measurements from being affected by sample self-heating effects due to the Joule effect. In Figure 2c, the complete measurement setup is shown: the different components (i.e., the cables, the connector box, and the cryostat) constitute a continuous Faraday cage to minimize EMI towards the samples inside the cryostat.

The resistance–temperature curves were acquired, with a rise step in temperature included between 1 mK/min and 5 mK/min.

### 3.1. Cryogenic Tests on Patterned Ti/Au Bilayer Samples

Resistance–temperature tests were performed on the two patterned samples (two-square geometry in Figure 1b), ATV10 and ATV11. The measured resistance values were normalized to the $R_{N}$, i.e., the resistance value measured immediately after the superconducting-to-normal transition occurs (for ATV10, $R_{N}$ = 0.23 Ω; for ATV11, $R_{N}$ = 0.54 Ω). The different $R_{N}$ values can arise from different wire-bonding positions on the patterned structure, since the two-square geometry has no dedicated pads for the bonding, and from the not yet fully optimized fabrication parameters.

**Figure 3:**
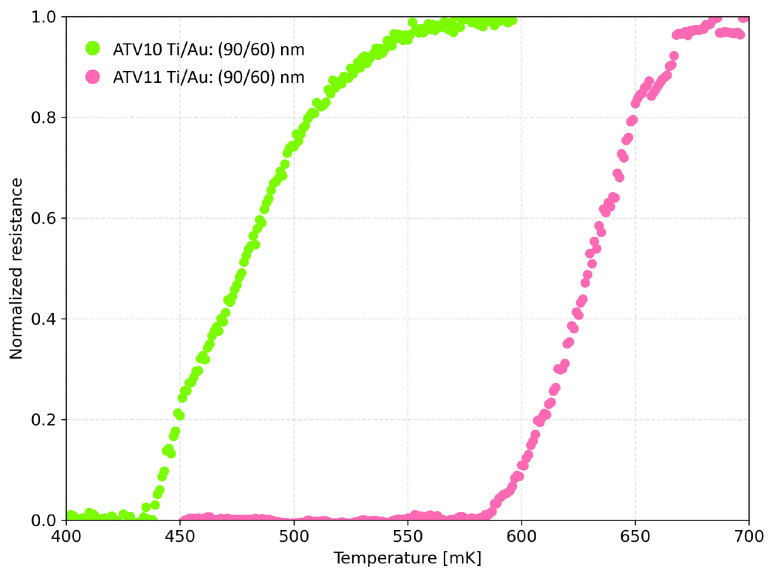
Resistance–temperature curves of the two-square geometry ATV10 (green left curve) and ATV11 (pink right curve) samples. The resistance is normalized with respect to the normal resistance values: for ATV10, $R_{N}$ is equal to 0.23 Ω; for ATV11, $R_{N}$ is equal to 0.54 Ω.

Figure 3 shows the results. A shift of about 150 mK was observed between the two curves. Although the thicknesses of the Ti/Au films are the same, such dissimilarity in behavior can be determined by slight differences in fabrication conditions (e.g., the initial RIE cleaning procedure). The broad transition width for both the samples can be attributed to an inhomogeneous titanium film, since the deposition process and conditions were not yet optimized. Moreover, as the two samples were tested after a different time interval since fabrication ending, part of the total shift (∼20 mK) is ascribed to the aging effect reported in the literature.

### 3.2. Cryogenic Tests on Non-Patterned Ti/Au Bilayer Samples

**Figure 4:**
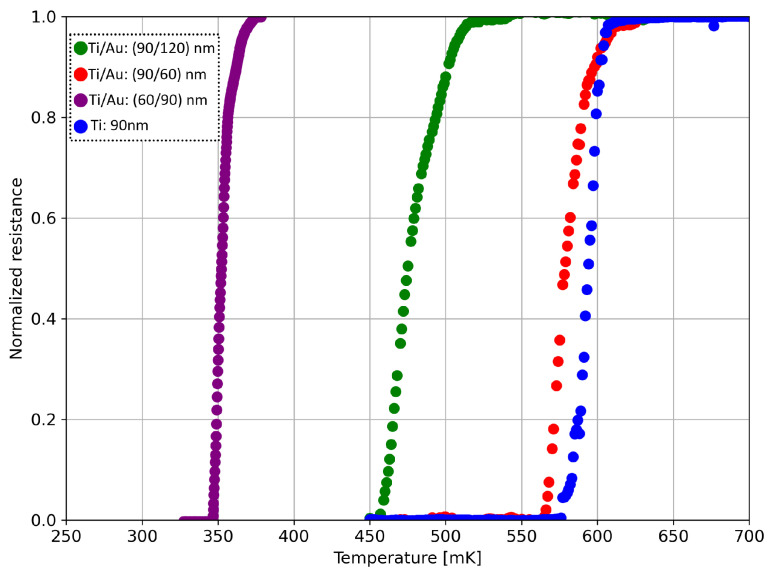
Normalized resistance–temperature curves for Ti/Au 90 nm/120 nm thick bilayer (green dots, Au/Ti ratio equal to 1.33, $R_{N}$= 0.11 Ω); Ti/Au 90 nm/60 nm thick bilayer (red dots, Au/Ti ratio equal to 0.57, $R_{N}$= 0.63 Ω); Ti/Au 60 nm/90 nm thick bilayer (purple dots, Au/Ti ratio equal to 1.5, $R_{N}$= 0.17 Ω); and Ti 90 nm thick monolayer (blue dots, $R_{N}$= 9.34 Ω).

Figure 4 plots a comparison of the results related to the first measurement run, with the sole Ti film acting as a reference.

The Ti/Au 90 nm/120 nm thick bilayer (green curve in Figure 4) presents a significant critical temperature reduction with respect to the Ti/Au 90 nm/60 nm thick bilayer (red in Figure 4). This behavior is consistent with the proximity effect theory, according to which an increase in the normal metal (Au) thickness leads to a strengthening of the proximity effect, i.e., a larger reduction in the titanium critical temperature [23]. Decreasing the titanium thickness from 90 nm (red curve in Figure 4) to 60 nm (purple curve in Figure 4), thus increasing the Au/Ti thickness ratio from 0.67 (red curve in Figure 4) to 1.5 (purple curve in Figure 4), leads to an even more pronounced shift in the critical temperature toward lower values.

## 4. Assessing Aging Effects

To characterize the effects of aging in such structures, multiple resistance–temperature measurements of the four non-patterned bilayer samples were performed at different time intervals from the conclusion of fabrication. In between every test, the samples were stored in two different environments: air environment at room temperature and vacuum environment inside the cryostat (vacuum level < $10^{-4}$ mbar).

### 4.1. Critical Temperature Shift

**Figure 5:**
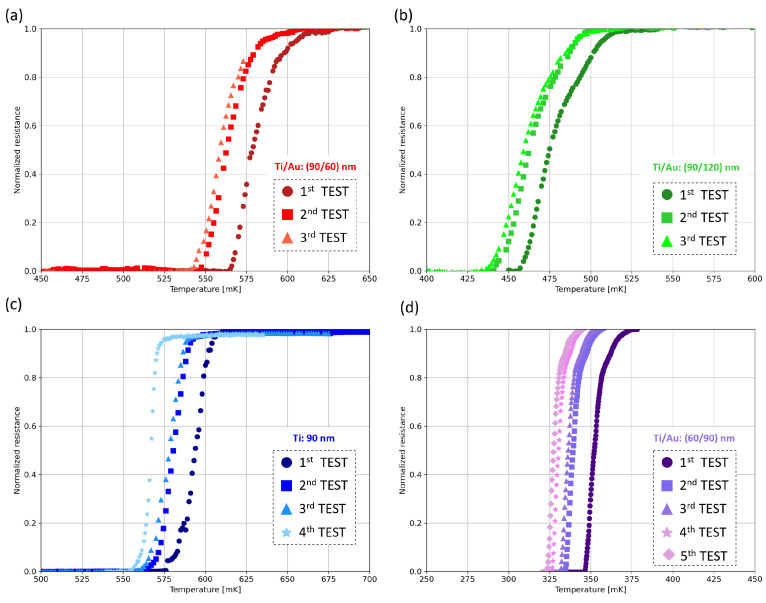
Resistance–temperature curves of Ti/Au bilayers measured after different time intervals: Ti/Au 90 nm/120 nm thick bilayer (**a**), Ti/Au 90 nm/60 nm thick bilayer (**b**), Ti/Au 60 nm/90 nm thick bilayer (**c**), and Ti 90 nm thick monolayer (**d**). The first test is performed 5 days after fabrication; the second test is performed 11 days after the first test (sample stored in vacuum for 6 days, then stored in air for 5 days); the third test is performed 10 after the second test (sample stored in vacuum for the whole 10 days); the fourth test is carried out 15 days after the third test (sample stored in vacuum for 13 days, then stored in air for 2 days).

The resistance–temperature characteristics of the four non-patterned samples at different time intervals from fabrication are reported in Figure 5.

The first test was performed 5 days after fabrication. The second test was performed 11 days after the first test, storing the samples for 6 days in vacuum and for 5 days in air to check the bilayers’ response to aging in a mixed environment. The samples were then stored in vacuum environment for 10 days before performing the third test. In all four samples, a degradation determined by aging caused the critical temperature to shift towards lower values. More specifically, the shift in the critical temperature values from the first to the third measurements is shown in Table 1.

To further investigate the aging trend, a fourth and a fifth test were performed on the 60 nm/90 nm thick sample, again changing the storage conditions. Specifically, the fourth test was performed 15 days after the third one, of which 13 days were spent in vacuum and 2 days in air, to assess the effect of re-exposure to the air environment. In this case, a ∼7 mK shift was registered. Finally, the fifth test was performed 17 days after the fourth test. During this time, the sample was stored 1 day in air and 16 days in vacuum. In this case, a smaller shift was reported (∼2.5 mK).

As a final note, the reported curve shift does not occur in combination with a change in the transition shape, i.e., the widening of the transition width.

Additionally, we observe that the shift value is similar for all the three fabricated bilayer samples, showing that the aging trend over time is independent from the thicknesses of the bilayer films, as further confirmed in Section 4.2.

### 4.2. Critical Temperature Degradation Model

**Figure 6:**
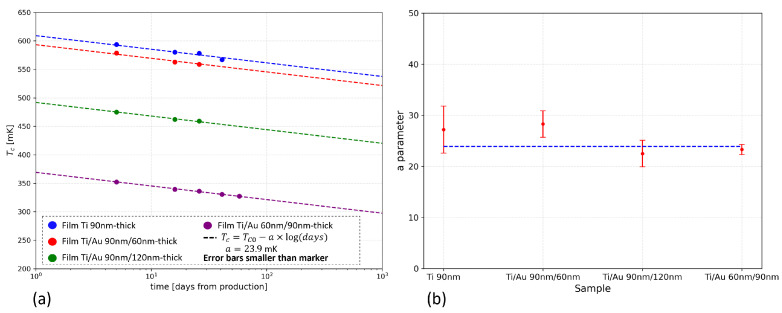
(**a**) Critical temperature ($T_{C}$) measured (colored dots) and evaluated (dashed lines) trends vs. time elapsed since sample fabrication, considering the days of storage in both air and vacuum environments. (**b**) Plot of the *a* parameter values obtained using a fitting procedure performed on the data acquired for the different samples. The error bars display the relative fitting errors.

The experimental data acquired on the fabricated samples are in agreement with the model proposed in [15] for describing the aging behavior of the Ti monolayer and Ti/Au bilayers for TES applications. The model expression that predicts the critical transition temperature $T_{C}$ as a function of time is [15]:

(1) $$T_{C}\left(t\right)=T_{C0}-a\times log\left(t\right),$$

where $T_{C0}$ is the critical transition temperature measured at the first test, *t* is the time in days, and *a* is a numerical parameter. Figure 6a reports the variation of $T_{C}$ over time (in days) for different bilayer samples considering the days spent both in vacuum and in air. The time axis is on a logarithmic scale. The $T_{C}$ value is taken as the temperature value at the 50% of the superconducting-to-normal transition curve (*R*($T_{C}$) = 0.5$R_{N}$).

The experimentally-obtained $T_{C}$ values, extracted from the tests described in Section 4.1, were fitted with Equation (1). The fitting procedure was performed independently for the different samples, recovering both the *a* parameter and the $T_{C0}$ values. The best fits are reported in Figure 6a (dashed lines). The *a* value is determined by averaging the results obtained using the fitting procedure on the data acquired for the different samples. The results of the fitting procedure for the *a* parameter are reported in Figure 6b, showing that there is not a statistically meaningful difference in the aging trend over time for the various bilayers, regardless of the Ti/Au layers’ thicknesses. The resulting average value of the parameter is *a* = (23.9 ± 0.9) mK. As we can see in Figure 6a, the experimental data show very good agreement with the theoretical behavior.

**Figure 7:**
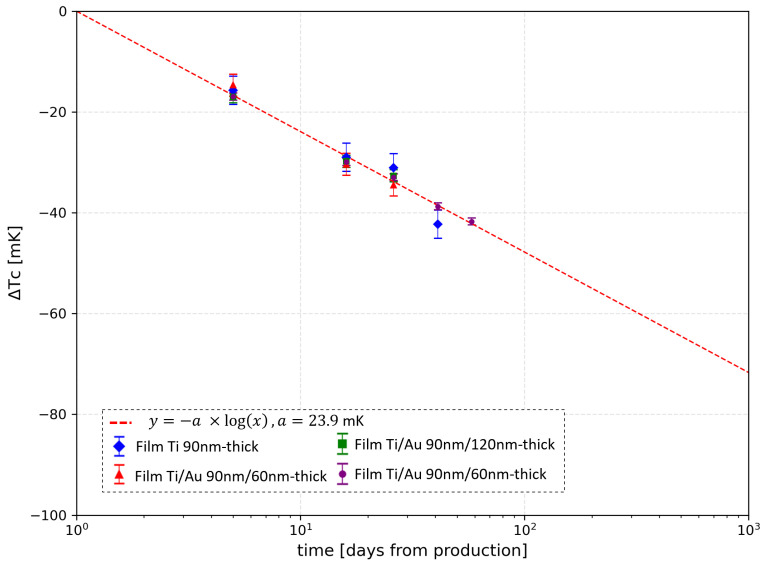
$\Delta T_{C}$ plot (colored dots) and evaluated trend (dashed lines) vs. time elapsed since sample fabrication. The error bars display the total error, taking into account the experimental error and the fitting error on $T_{C0}$.

To better compare the data and the theoretical behavior, Figure 7 reports the difference $\Delta T_{C}$ between the critical transition temperature measured at a certain time $T_{c}\left(t\right)$ and its initial values $T_{C0}$ (assessed by the previous fit analysis) for all the different samples. For each point, the error takes into account both the estimated experimental error and the fitting error on the $T_{C0}$ values. The experimental error is equal to 1.8 mK, and it is obtained considering the 4-wire setup error and the intrinsic error of the reading due to the calibration of the cryostat thermometer. The experimental data show very good agreement with the theoretical model.

### 4.3. Discussion

The degradation over time of the critical temperature for TES bilayers has been reported in several studies [16,24], whereas the origin of such phenomenon has not yet been exhaustively investigated due to the complexity of cryogenic tests.

The contamination of the Ti film by oxygen molecules has been suggested as one of the possible causes of degeneration [14,15]. This is particularly true in the case of Ti films that are not covered with Au, as they are subjected to oxidation due to their direct exposure to the environment [15]. In the case of Ti/Au bilayers, such a phenomenon may involve oxygen molecules from the environment in the evaporation chamber trapped in the Ti film during the deposition [25].

The decrease in the critical temperature has also been explained as a consequence of a change in the Ti–Au interface properties, due to a diffusive process between Ti and Au at the interface. In particular, Ti diffuses inside Au grain boundaries, leading to a thinning of the titanium layer [16]. Also, the formation of a Ti–Au mixed interlayer, a consequence of the diffusion process, can affect the interface quality, resulting in a change in the critical temperature [19].

**Figure 8:**
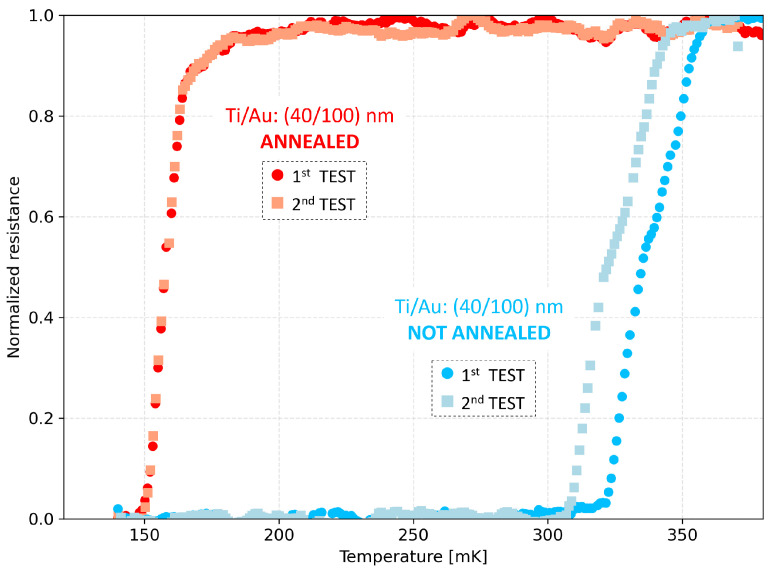
Normalized resistance–temperature curves measured for non-annealed (blue and light blue, $R_{N}$ = 0.15 Ω) and annealed (red and orange, $R_{N}$ = 0.15 Ω) Ti/Au 40 nm/100 nm thick bilayers. The second test is performed 7 days after the first test (sample stored in air for 3 days and in vacuum for 4 days).

To stabilize the TES critical temperature, an annealing treatment is reported in the literature as an effective method [15]. Thus, an annealing procedure was tested on a Ti/Au 40 nm/100 nm thick sample. The process was carried out in air environment at 150 °C for 24 h using a Memmert UM 200 (Memmert GmbH + Co. KG, Schwabach, Germany) oven. To avoid thermal shock, the sample underwent a temperature ramp during both the heating and the cooling phases. After annealing, two R–T tests were carried out one week apart (the first measurement in red and the second one in orange). Figure 8 plots a comparison between the annealed Ti/Au 40 nm/100 nm thick sample (red and orange in the figure) and a non-annealed Ti/Au 40 nm/100 nm thick sample (blue and light blue in the figure) fabricated with the same procedure in the same fabrication run. It is clearly visible that the annealed sample shows a net decrease in the critical temperature, probably as a result of a Ti–Au mixed layer at the interface between the two films due to the high temperatures reached. Moreover, the non-annealed sample shows aging after one week, with a change in its $T_{C}$ of ∼15 mK as expected from the above-reported analysis (see Section 4.2), whereas the annealed sample shows a stable R–T curve on the same time interval.

## 5. Conclusions

We are setting up a new fabrication line of TES detectors to be used as cryogenic anticoincidence devices. In this work, we report the development of Ti/Au bilayers for TES detectors, aiming to achieve a stable sensing performance with respect to aging effects. For this purpose, patterned and non-patterned Ti/Au bilayer samples were built using microfabrication technology processes. Cryogenic resistance–temperature characteristics were measured at different time intervals ranging from 5 to 60 days after the end of fabrication in order to assess the aging effect on samples with different Ti/Au thicknesses. In doing so, we expanded the data already available in the literature, demonstrating that the aging effect and its trend over time do not depend on the thickness of the bilayer films. Moreover, as reported by other groups [15], exposing samples to an annealing treatment in air at 150 °C for 24 h as the final fabrication step assures the stabilization of the critical temperature over time.

Further studies are planned to optimize the stabilization process and to achieve a reliable fabrication procedure for the patterned samples. The presented outcomes are promising steps towards the development of stable and efficient TES detectors for space and particle physics applications.

## Figures and Tables

**Table 1:** Critical temperature values obtained in the first, second, and third tests for the samples: Ti/Au 90 nm/60 nm thick, Ti/Au 90 nm/120 nm thick, and Ti/Au 60 nm/90 nm thick.

Sample	$T_{C1}$	$T_{C2}$	Total Days in Between	Days in Vacuum	Days in Air
Ti/Au 90 nm/60 nm thick	578 mK	563 mK	11	6	5
Ti/Au 90 nm/120 nm thick	475 mK	462 mK	11	6	5
Ti/Au 60 nm/90 nm thick	352 mK	340 mK	11	6	5
Ti 90 nm thick	594 mK	580 mK	11	6	5
**Sample**	$T_{C2}$	$T_{C3}$	**Total Days in Between**	**Days in Vacuum**	**Days in Air**
Ti/Au 90 nm/60 nm thick	563 mK	559.5 mK	10	10	0
Ti/Au 90 nm/120 nm thick	462 mK	459 mK	10	10	0
Ti/Au 60 nm/90 nm thick	340 mK	338 mK	10	10	0
Ti 90 nm thick	580 mK	577.5 mK	10	10	0

## Data Availability

Data are contained within the article.
